# The effect of citrus essential oil encapsulation on antioxidant capacity and bacterial growth in a milk matrix during storage

**DOI:** 10.1002/jsfa.70344

**Published:** 2025-11-20

**Authors:** Feilong Yang, Vincenzo Fogliano, Ashkan Madadlou

**Affiliations:** ^1^ Food Quality and Design Group, Department of Agrotechnology and Food Sciences Wageningen University and Research Wageningen The Netherlands; ^2^ Institute of Urban Agriculture, Chinese Academy of Agricultural Sciences Chengdu National Agricultural Science and Technology Center Chengdu China; ^3^ School of Food and Nutritional Sciences University College Cork Cork Ireland

**Keywords:** antibacterial ability, antioxidant ability, citrus essential oil, encapsulation, food matrix, zein particles

## Abstract

**BACKGROUND:**

The functionality of food can be improved by supplementing it with bioactive substances. However, the interactions between these substances and food components can compromise their functional efficacy. This study investigated the potential to reduce functional losses by encapsulating citrus essential oil (CEO) in zein particles (ZPs). We examined the antibacterial and antioxidant efficacy of these encapsulates at different CEO release rates in skimmed and whole milk samples. Slow CEO‐releasing ZPs were developed by coating CEO‐loaded ZPs with calcium phosphate and alginate gel (CaP‐AlgGel‐CEO/ZPs), while fast CEO‐releasing ZPs served as controls.

**RESULTS:**

The solubilization of CEO was enhanced in milk probably by hydrophobic interactions and colloidal stabilization provided by milk components. This increased solubilization of CEO facilitated its release from CEO encapsulates. Free CEO exhibited no DPPH scavenging activity when dissolved in milk. In contrast, CEO encapsulates, especially those with a slow‐release rate, demonstrated significant DPPH scavenging activity. In comparison with the fast‐release counterparts, the slower CEO release caused greater bacterial inhibition. Principal component analysis revealed that the type of matrix influenced the antioxidant capacity of supplemented bioactive substances, while storage time affected their antibacterial effectiveness.

**CONCLUSION:**

This study underscores the advantages of encapsulation techniques in preserving the functional efficacy of bioactive substances by mitigating adverse interactions with food components. © 2025 The Author(s). *Journal of the Science of Food and Agriculture* published by John Wiley & Sons Ltd on behalf of Society of Chemical Industry.

## INTRODUCTION

Citrus essential oil (CEO) is a volatile mixture of hydrophobic terpenoids extracted from citrus peels. As secondary metabolites, these compounds not only possess pleasant aroma but also exhibit antioxidant, antibacterial, and anti‐inflammatory properties. This makes them popular as food additives for preservation and flavor enhancement.^1^, ^2^ Despite these advantages, the physicochemical properties of CEO present certain challenges for its application. The high hydrocarbon terpene content of CEO makes it highly hydrophobic and volatile, as well as susceptible to degradation by free radicals, significantly limiting its effectiveness in aqueous food systems.^3^

Encapsulation has been extensively used for improving the dispersibility of essential oils within aqueous food matrices.^4^ Furthermore, encapsulated shells protect essential oils from environmental factors, resulting in slower degradation and reduced evaporation during storage.^5^ The improvements in physical properties, in turn, enhance the antibacterial and antioxidant efficacy of essential oils, making them more effective for food preservation.^6^ For example, essential oils encapsulated within nano‐emulsions or nanoparticles have demonstrated superior antibacterial activity, attributed to the amphiphilic nature of the components and the nanoscale size, which facilitate easier transportation through bacterial cell membranes.^7^, ^8^, ^9^ In addition, due to improved dispersibility and a higher surface‐area‐to‐volume ratio within food matrices, encapsulation of essential oils increased their antioxidant activity compared to their free counterparts.^9^, ^10^

Controlled release of encapsulated essential oils into food matrices is a valuable approach for preserving and enhancing food quality. In this strategy, essential oils exhibit their bioactive effects once they are released and dissolved within the food matrix.^11^ However, these effects may be diminished due to undesired interactions between essential oils and matrix components. It has been reported that the antimicrobial activity of *Baccharis dracunculifolia* DC essential oil is lower in whole milk compared to skimmed milk.^12^ The food matrix components, particularly proteins and lipophilic substances, can interact with active substances present in essential oils, thereby diminishing their antimicrobial activity towards pathogenic bacteria.^12^, ^13^

In our previous study, we encapsulated CEO within zein particles (ZPs) and achieved slow‐release profiles through inorganic coating and biopolymeric networking.^14^ Among the formulations, calcium phosphate‐coated and alginate‐gel‐networked ZPs exhibited the highest CEO retention, owing to their compact layered structures. However, the functional performance of these CEO‐loaded encapsulates in simulated or real food systems remained unexplored. This study aims to evaluate whether the encapsulation technique enhances the functionality of CEO in food matrices. We added CEO encapsulates with different release rates to water and milk of different fat contents and monitored the temporal changes in CEO distribution and functional properties of the mixtures. By correlating these release profiles with antioxidant and antibacterial activities of the mixtures, we seek to identify the key factors governing CEO functionality, thereby facilitating the rational application of the CEO‐loaded particles in food products.

## MATERIALS AND METHODS

### Materials

Pasteurized skimmed milk (fat (*w/w*) < 1%) and whole milk (fat (*w/w*) = 13%) were obtained from a local supermarket (Jumbo, The Netherlands). Zein (Z3625) and CEO (orange oil, W282510) were purchased from Sigma‐Aldrich (Shanghai, China). Sodium alginate was obtained from Sigma‐Aldrich (Merck KGaA, Darmstadt, Germany). Plate count agar was purchased from Merck (Schiphol‐Rijk, The Netherlands) and peptone physiological salt solution was procured from Tritium Microbiologie BV (Eindhoven, The Netherlands). Sodium dihydrogen phosphate (NaH_2_PO_4_), disodium phosphate (Na_2_HPO_4_), calcium chloride dihydrate (CaCl_2_·2H_2_O), and ethanol were purchased from Sigma‐Aldrich Chemie B.V. (Wageningen, The Netherlands). All chemicals used were of analytical or chromatographic grade.

### Particle preparation

ZPs with different CEO‐releasing rates were prepared following a previously reported procedure.^14^ Zein (150 mg) and CEO (30 mg) were dissolved in 10 mL of 85% ethanol–water (*v/v*) solution. This was followed by adding four times volume (40 mL) of phosphate buffer (10 mmol L^−1^, pH 7.5) into the solution. The resulting solution, which turned opaque, was dialyzed against phosphate buffer (10 mmol L^−1^, pH 7.5) at 4 °C for 20 h to remove ethanol. The resulting sample was CEO‐loaded ZPs in phosphate buffer (10 mmol L^−1^, pH 7.5) (abbreviated as CEO/ZPs). Subsequently, 1 mL of sodium alginate solution (5 mg mL^−1^) was added into 10 mL of CEO/ZPs dispersion, forming alginate‐supplemented ZPs (abbreviated as Alg‐CEO/ZPs). This was followed by supplementing the dispersion with 100 μL of 4 mol L^−1^ CaCl_2_ solution and stirring for 1 min, forming calcium phosphate‐coated and alginate gel‐networked ZPs (abbreviated as CaP‐AlgGel‐CEO/ZPs). Both Alg‐CEO/ZPs and CaP‐AlgGel‐CEO/ZPs dispersions were freshly prepared before starting the storage experiment.

### CEO encapsulation and release

d‐Limonene, the main component of CEO, was used as reference of CEO when determining CEO content. Gas chromatography (GC, Agilent 5975C; Agilent Technologies, Santa Clara, CA, USA), equipped with HP‐5 capillary column (100 m × 0.25 mm inner diameter (i.d.), film thickness, 0.25 μm) and flame ionization detector, was used for d‐limonene content measurements.^15^

#### Encapsulation efficiency

Encapsulation efficiency (%) of CEO in ZPs was calculated by dividing encapsulated CEO content after particle preparation with the amount of CEO initially added into the aqueous‐ethanol solution.

#### Residual encapsulated CEO in ZPs

The amount of the CEO remained encapsulated within ZPs during storage of CEO encapsulate‐supplemented matrix was measured as follows: 25 mL of CEO encapsulates (i.e., CEO/ZPs, Alg‐CEO/ZPs, and CaP‐AlgGel‐CEO/ZPs) was mixed with 25 mL of food matrix (i.e., water, skimmed milk, and whole milk); the mixture was supplemented with sodium azide, and stored for 3 days within polypropylene plastic tubes at 25 °C. Next, 10 mL of CEO encapsulate‐supplemented matrix was sampled from the middle height of the tubes at various time points over the storage period. The tubes were slightly agitated before sampling. The sampled aliquots were supplemented with 1 mL of 0.5 mol L^−1^ sodium chloride (NaCl) solution to facilitate the precipitation of ZPs, followed by vacuum filtration through a filter paper with a pore size of 3 μm. The filtration residue was extracted with 10 mL of *n*‐hexane for CEO content measurement by GC. The residual encapsulated CEO as percentage was calculated by the following formula:

$$\text{Residual encapsulated}\;\mathrm{CEO}\;\left(\%\right)=\begin{array}{l} \frac{\text{The}\;\mathrm{CEO}\;\text{content measured in the filtration residue}\;\mathrm{at}\;\text{each sampling time}}{\text{The}\;\mathrm{CEO}\;\text{content measured in the filtration residue}\;\mathrm{at}\;\text{time}\;0\;\text{of storage}}\times 100 \end{array}$$

The values of residual encapsulated CEO (%) at different time points were fitted to Korsmeyer–Peppas (Eqn (1)), first‐order (Eqn (2)) and Higuchi (Eqn (3)), models to investigate the kinetics of CEO release from ZPs:

(1) $$\text{Residual encapsulated}\;\mathrm{CEO}\;\left(\%\right)=\left(1\;-\;k\;\times \;t^{n}\right)\times 100$$

(2) $$\text{Residual encapsulated}\;\mathrm{CEO}\;\left(\%\right)=\frac{a\;\times \;e^{-\mathrm{kt}}+b}{100}\times 100$$

(3) $$\text{Residual encapsulated}\;\mathrm{CEO}\;\left(\%\right)=\left(1-\;\frac{k\;\times \;\sqrt{t}}{100}\;\right)\times 100$$

where *t* is the sampling time in days; *k* is the rate constant of CEO release; *n* is the release exponent of Korsmeyer–Peppas model; *a* and *b* are parameters of the first‐order model fitted for the initial and final values of residual encapsulated CEO (%), respectively.

#### Dissolved CEO in food matrix

The amount of the CEO that dissolved into the food matrix was measured as follows: For CEO encapsulates, CEO that dissolved into the food matrix was calculated by subtracting residual encapsulated CEO content from total CEO content. Total CEO content, which contained dissolved CEO in food matrix and residual encapsulated CEO in ZPs, was determined according to the procedure outlined in ‘Residual encapsulated CEO in ZPs’ section, albeit without any filtration steps. It is worth noting that the initially added CEO content was not used as the value of the total CEO content because CEO could be lost by evaporating into the air, floating on the surface of the solution, and adhering to the walls of the tubes during the storage, which causes the varying value of total CEO content during storage. The percentage of dissolved CEO in food matrix was calculated by the following formula:

$$\text{Dissolved}\;\mathrm{CEO}\left(\%\right)=\begin{array}{l} \frac{\text{Total}\;\mathrm{CEO}\;\text{content}\;\mathrm{at}\;\text{each sampling time}}{\text{Total}\;\mathrm{CEO}\;\text{content}\;\mathrm{at}\;\text{time}\;0\;\text{of storage}}\\ \times \;100-\text{Residual encapsulated}\;\mathrm{CEO}\;\text{percentage} \end{array}$$

where the percentage difference was used instead of the content difference to eliminate methodological errors arising from the different measurement procedures for total CEO and residual encapsulated CEO.

For free CEO, dissolved CEO in food matrix was measured as in the earlier procedure, but the supplemented CEO encapsulates were replaced by free CEO (the amount equaled to the average of encapsulated CEO content in 25 mL of the three types of CEO encapsulates at time 0) and deionized water. Residual encapsulated CEO (%) was zero for free CEO‐supplemented food matrix. Thus, dissolved CEO (%) in free CEO‐supplemented food matrix was calculated by the following formula:

$$\begin{array}{l} \text{Dissolved}\;\mathrm{CEO}\left(\%\right)\;\text{in}\;\text{free}\;\mathrm{CEO}\;\text{supplemented food matrix}\\ \;=\frac{\text{Total}\;\mathrm{CEO}\;\text{content}\;\mathrm{at}\;\text{each sampling time}}{\text{Total}\;\mathrm{CEO}\;\text{content}\;\mathrm{at}\;\text{time}\;0\;\text{of storage}}\;\times \;100 \end{array}$$

### Antioxidant activity

The DPPH (2,2‐diphenyl‐1‐picrylhydrazyl) radical scavenging activity (%) of food matrix (i.e., water, skimmed milk, and whole milk) before or after supplementing free CEO or CEO encapsulates was determined according to a reported methodology.^16^ Briefly, 1 mL of CEO encapsulate‐supplemented food matrix, which was periodically sampled as the aforementioned procedures, was mixed with 3 mL of 200 μmol L^−1^ DPPH in ethanol. The mixture was vortexed vigorously and incubated in darkness at room temperature for 30 min, followed by filtration using syringe filters (polyvinylidene fluoride (PVDF), 0.45; Millipore, Billerica, MA, USA). The absorbance of the filtrate (Af) was measured at 517 nm using aqueous ethanol solution as the blank (Ab). The percentage of DPPH radical scavenging activity (%) was calculated by the following formula:

$$\text{DPPH radical scavenging activity}\;\left(\%\right)=\frac{\mathrm{Ab}\;-\;\mathrm{Af}}{\mathrm{Ab}}\;\times \;100$$

The antioxidant activity of CEO additives (i.e., free CEO or CEO encapsulates) in food matrix is indicated by the variation in DPPH radical scavenging activity (%) of the matrix before and after supplementing free CEO or CEO encapsulates as presented by the following formula:

$$\text{The antioxidant activity}\left(\%\right)=\begin{array}{l} \text{DPPH radical scavenging activity}\left(\%\right)\;\text{of the matrix after supplementation}\\ -\;\text{DPPH radical scavenging activity}\left(\%\right)\;\text{of the matrix before supplementation} \end{array}$$

The higher value of the antioxidant activity, the more DPPH radicals CEO additives can scavenge. Therefore, 100% of the antioxidant activity means all the added DPPH radicals have been scavenged.

### Enumeration of bacteria

The enumeration of total aerobic mesophilic bacteria was performed using the pour plate method.^11^ CEO or CEO encapsulates were mixed with food matrix without adding sodium azide, which allowed the normal growth of bacteria in the pasteurized milk matrices and deionized water. This was followed by sampling at different timepoints over a storage duration of 3 days. The bacterial population was determined by serially diluting the sampled mixture ten‐fold with peptone physiological salt solution. A 100 μL of the diluted aliquots were brought onto plate count agar. The plates were incubated at 20 °C for 24 h before counting the bacterial colonies.

A modified Gompertz model was used to fit the bacterial growth curves.^17^ The formula described the total viable count of bacteria as a function of time was as follows:

$$\ln\frac{N}{N_{0}}=A_{s}\;\times \;\exp\;\left(-\exp\left(\frac{\mu_{\max}e}{A_{s}}\left(\lambda -t\right)+1\right)\right)$$

Herein, *A*_*s*_ is the natural logarithm of the asymptotic value of relative population size (ln(CFU mL^−1^)), *μ*_max_ is the maximum specific growth rate (in d^−1^), *λ* is the duration of the lag phase (in days), *t* is the storage time (in days), and $\frac{N}{N_{0}}$ is the ratio of the microbial cell density (in CFU mL^−1^) to the initial density value.

### Statistical analysis

All data were reported as mean ± SD (standard deviation, indicated by error bars). R package nlstools (tools for non‐linear least squares fit) is open‐source and provides extensive diagnostic capabilities compared to other tools.^18^ The first order and Gompertz model were fitted using nlstools and the significant differences of release constants were determined when *P* < 0.05. Principal component analysis (PCA) was conducted using R package stats.

## RESULTS AND DISCUSSION

### Release kinetics of CEO/ZPs in different matrices

**Figure 1:**
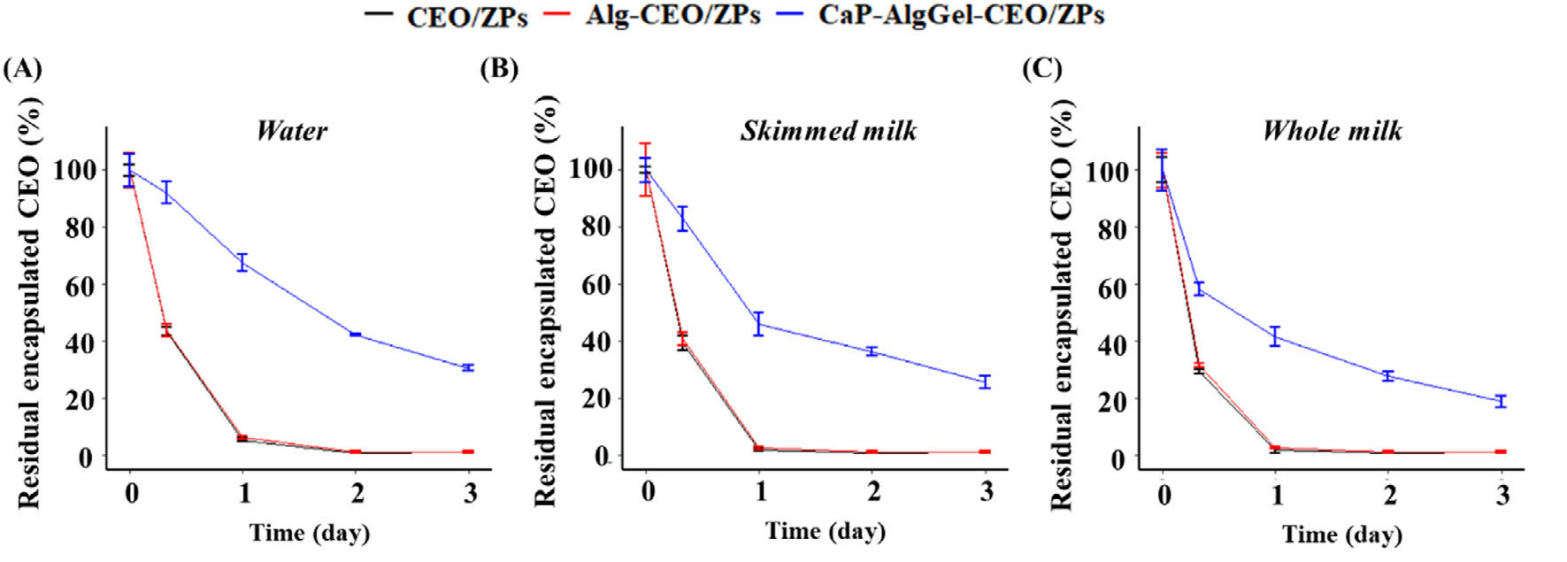
Changes in the percentage of CEO remained encapsulated during 3 days of storage after adding CEO encapsulates into (A) water, (B) skimmed milk, and (C) whole milk. CEO/ZPs: citrus essential oil‐encapsulated zein particles; Alg‐CEO/ZPs: alginate‐supplemented CEO/ZPs; CaP‐AlgGel‐CEO/ZPs: calcium phosphate‐coated and alginate gel‐networked CEO/ZPs.

The encapsulation efficiency of the different types of CEO encapsulates was 69.2% for CEO/ZPs, 67.5% for Alg‐CEO/ZPs, and 62.3% for CaP‐AlgGel‐CEO/ZPs in line with our previous work.^14^ Across all forms of capsules, CEO was rapidly released from both CEO/ZPs and Alg‐CEO/ZPs, with nearly no encapsulated CEO remaining after the first day. In contrast, CaP‐AlgGel‐CEO/ZPs exhibited a sustained slow release of CEO throughout the entire storage period (Fig. 1(A)–(C)). To further elucidate the release behavior, three kinetic models were applied to the time‐dependent residual encapsulated CEO (expressed as a percentage of the initially added CEO). The Korsmeyer–Peppas and first‐order models showed high coefficient of determination (*R* ^2^) values across all release scenarios, whereas the Higuchi model did not provide a good fit (Table 1). The release exponent (*n*) derived from the Korsmeyer–Peppas model reflected the transport mechanism of CEO release.^19^ For CaP‐AlgGel‐CEO/ZPs in water, *n* was 0.77, indicating that CEO release was governed by the relaxation of polymeric coatings. In contrast, CEO/ZPs and Alg‐CEO/ZPs in water exhibited *n* values below 0.5, suggesting a pseudo‐Fickian release process.^20^ Although pseudo‐Fickian diffusion resembles classical Fickian diffusion, it typically reaches release equilibrium faster than an ideal Fickian process, the diffusion‐dominated release. Relative to water, both skimmed milk and whole milk yielded lower *n* values, indicating a shift toward more pseudo‐Fickian, notably for CaP‐AlgGel‐CEO/ZPs. We attribute this to interactions with milk proteins and casein micelles, as well as partitioning of CEO into fat globules which together alter diffusion pathway and thus observed kinetics.

**Table 1:** Release parameters (*k*, *n*, and adjusted *R* ^2^) of Korsmeyer–Peppas and first‐order models fitted to time‐dependent residual encapsulated citrus essential oil (CEO) percentages

		Water			Skimmed milk				Whole milk		
	CEO/ZPs	Alg‐CEO/ZPs	CaP‐AlgGel‐CEO/ZPs		CEO/ZPs	Alg‐CEO/ZPs	CaP‐AlgGel‐CEO/ZPs		CEO/ZPs	Alg‐CEO/ZPs	CaP‐AlgGel‐CEO/ZPs
*Korsmeyer–Peppas model*											
*k*	0.83 ± 0.03	0.83 ± 0.03	0.31 ± 0.03		0.86 ± 0.03	0.85 ± 0.03	0.45 ± 0.03		0.89 ± 0.02	0.88 ± 0.02	0.59 ± 0.02
*n*	0.22	0.22	0.77		0.19	0.20	0.50		0.14	0.15	0.30
Adjusted *R* ^2^	95.64%	94.47%	97.57%		95.40%	92.00%	95.23%		96.92%	96.06%	96.08%
*First‐order model*											
*k*	2.61 ± 0.12^bc^	2.59 ± 0.25^bc^	0.42 ± 0.17^d^		2.93 ± 0.16^b^	2.82 ± 0.41^bc^	1.05 ± 0.23^d^		3.77 ± 0.27^a^	3.56 ± 0.33^a^	1.83 ± 0.41^c^
Adjusted *R* ^2^	99.65%	98.45%	98.32%		99.52%	96.62%	97.32%		99.23%	98.63%	94.93%
*Higuchi model*											
*k*	69.42 ± 3.96	69.22 ± 4.02	37.81 ± 2.15		70.37 ± 4.42	70.06 ± 4.58	44.70 ± 1.94		71.27 ± 5.06	70.88 ± 4.91	51.45 ± 2.30
Adjusted *R* ^2^	79.77%	79.30%	95.79%		75.13%	74.03%	95.59%		67.31%	69.09%	91.81%

*Note*: The superscript letters for *k* values in a row indicate the significant differences (*P* < 0.05). CEO/ZPs: CEO‐encapsulated zein particles (ZPs); Alg‐CEO/ZPs: alginate‐supplemented CEO/ZPs; CaP‐AlgGel‐CEO/ZPs: calcium phosphate‐coated and alginate gel‐networked CEO/ZPs.

**Figure 2:**
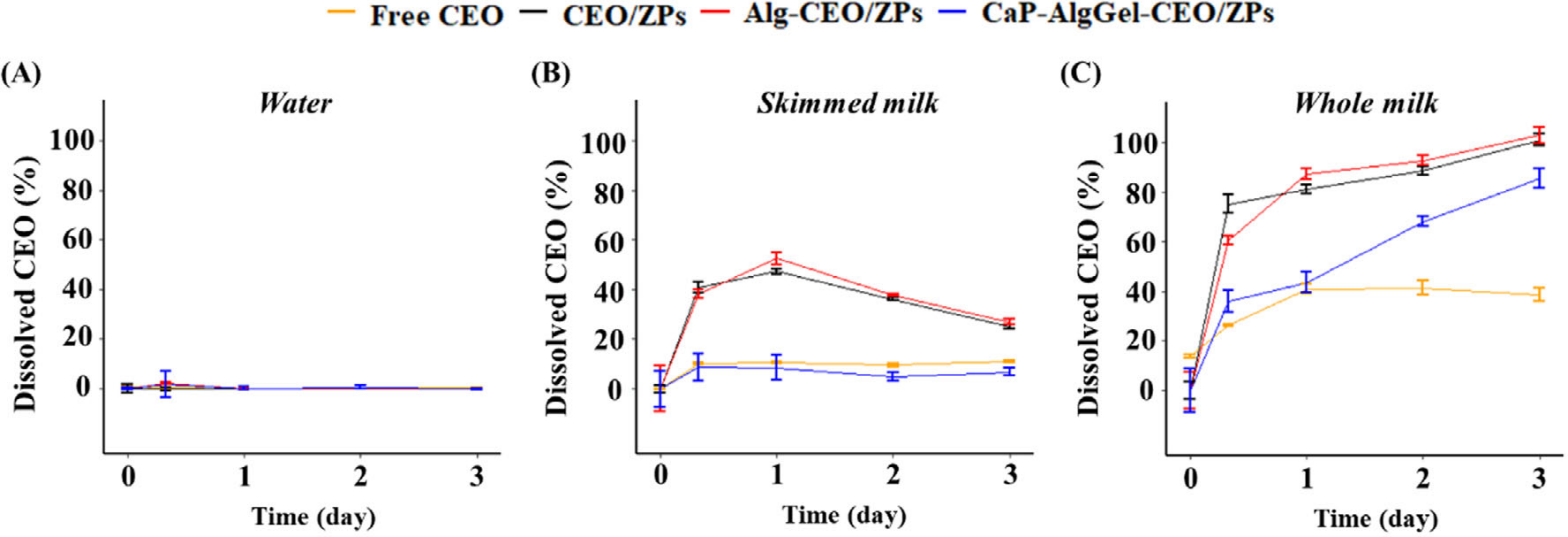
Changes in the percentage of dissolved CEO after adding free CEO or CEO encapsulates into (D) water, (E) skimmed milk, and (F) whole milk during 3 days of storage. CEO/ZPs: CEO‐encapsulated ZPs; Alg‐CEO/ZPs: Alginate‐supplemented CEO/ZPs; CaP‐AlgGel‐CEO/ZPs: Calcium phosphate‐coated and alginate gel‐networked CEO/ZPs.

The first‐order model provided a good fit across all encapsulation types and food matrices, enabling a direct comparison of release rates through their rate constants (*k*). Specifically, whole milk resulted in a faster release of CEO from ZPs compared to skimmed milk and water, as indicated by the higher rate constant (*k*) of a given type of CEO encapsulate in three matrices (Table 1). This can be ascribed to the higher solubility of CEO in whole milk compared to skimmed milk and water (Fig. 2(A)–(C)), driving the diffusion of CEO out of the encapsulate. Furthermore, residual encapsulated CEO of CaP‐AlgGel‐CEO/ZPs was significantly higher than those of CEO/ZPs and Alg‐CEO/ZPs (*P* < 0.05) over the storage period, with the release rate constant (*k*) of CaP‐AlgGel‐CEO/ZPs significantly lower than the others (Table 1). This finding aligned with our previous results, which demonstrated that CaP‐AlgGel‐CEO/ZPs exhibited a slower release rate of CEO compared to non‐coated CEO encapsulates. It further showed this trend was consistent across all types of food matrices.

### Distribution of encapsulated CEO in the matrices

Figure 2(A)–(C) shows the amount of dissolved CEO in the matrices (expressed as the percentage of initially added CEO content) after supplementing free CEO or CEO encapsulates into the matrices. The CEO released from the ZPs can dissolve in food matrix, with the extent of solubilization influenced by the polarity compatibility and emulsification ability of food matrix.^21^, ^22^ The undissolved CEO evaporated into the air, floated on the surface of the solution, and adhered to the walls of the tubes during the storage, becoming undetectable by applied measurement. This was indicated by Fig. 2(A) where the amount of dissolved CEO was zero for all types of CEO additive. The ultra‐low solubility of d‐limonene, the main component of CEO, in water has been described in the literature.^23^ In contrast, CEO gradually dissolved in milk matrices as it released from all three CEO encapsulates (i.e., CEO/ZPs, Alg‐CEO/ZPs, and CaP‐AlgGel‐CEO/ZPs) (Fig. 2(A)–(C)). This was due to the large amount of hydrophobic components, including fat granules and proteins present in milk matrices. Fats can increase the solubilization of hydrophobic substances like limonene (which constitutes 95% of CEO) with a log*P* value of 4.552,^24^ while proteins have hydrophobic regions capable of adsorbing CEO.^25^ The solubilization of CEO was more evident in whole milk than in skimmed milk as shown in a given type of CEO additive in milk matrices (Fig. 2(B),(C)). The difference in solubility between skimmed milk and whole milk (*P* < 0.05) is due to different fat contents in the two milks. The fat in whole milk can serve as a reservoir to dissolve CEO.^22^

It is possible that the milk components act as colloidal stabilizers for CEO droplets, further enhancing CEO solubility/dispersibility. Both caseins and whey proteins serve as emulsifiers, rapidly adsorbing at oil–water or air–water interfaces and forming a protective film around oil droplets or air bubbles.^26^ This phenomenon was pronounced in CEO encapsulate‐supplemented milk matrices. As shown in Fig. 2(A)–(C), the amount of dissolved CEO over a duration of 3 days was significantly higher in CEO encapsulate‐supplemented milk matrices than the free CEO counterpart, except for CaP‐AlgGel‐CEO/ZPs‐supplemented milk matrices. The higher CEO solubilization in CEO encapsulate‐supplemented milk matrices suggested that the CEO released from ZPs could adsorb onto the surface of particles, where it was stabilized by milk proteins,^26^ allowing it to be retained in the matrices. However, the stabilization ability of proteins in skimmed milk was not enough to fully keep CEO in dispersion over the course of 3 days, as evidenced by the gradual decrease in dissolved CEO percentages after day 1 (Fig. 2(B)). CaP‐AlgGel‐CEO/ZPs‐supplemented milk matrices (especially for skimmed milk) stood out as an exception, showing a low content of dissolved CEO, similar to that in free CEO–skimmed milk mixture (Fig. 2(B)). This can be attributed to the slow release of CEO from CaP‐AlgGel‐CEO/ZPs, which resulted in a reduced amount of CEO available for solubilization. Additionally, the limited surface area of the CaP‐AlgGel‐CEO/ZPs particle aggregates^14^ restricts the adsorption of the released CEO.

### The antioxidant activity of CEO encapsulates in matrices

**Figure 3:**
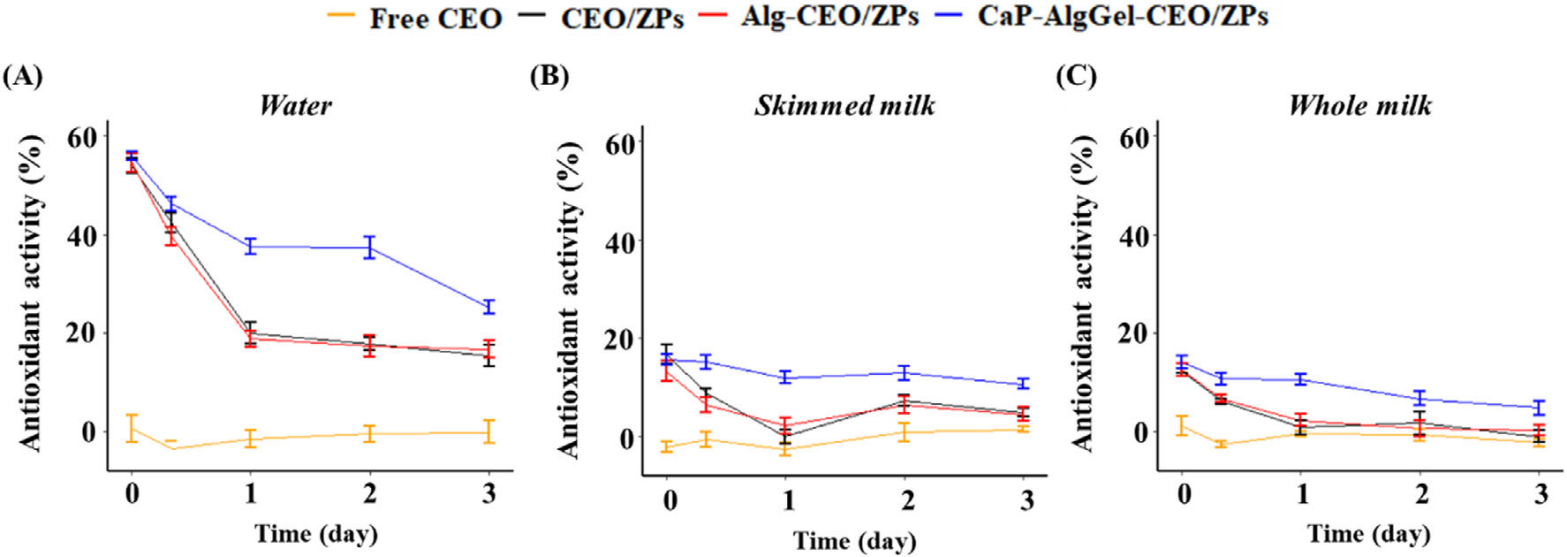
The antioxidant activity of free CEO or CEO encapsulates added to food matrices during storage for 3 days. The matrices were (A) water, (B) skimmed milk, and (C) whole milk, respectively. CEO/ZPs: citrus essential oil‐encapsulated zein particles; Alg‐CEO/ZPs: alginate‐supplemented CEO/ZPs; CaP‐AlgGel‐CEO/ZPs: calcium phosphate‐coated and alginate gel‐networked CEO/ZPs.

The antioxidant activity of free CEO or CEO encapsulates in food matrices was assessed by comparing the DPPH scavenging activity of matrices before and after mixing with these CEO additives. In water, as shown in Fig. 3(A), the antioxidant activity of CEO additives appeared to be closely associated with release rates of CEO encapsulates. The slow CEO‐releasing ZPs (i.e., CaP‐AlgGel‐CEO/ZPs) exhibited the highest antioxidant activity throughout the storage period, while the fast CEO‐releasing ZPs (i.e., CEO/ZPs and Alg‐CEO/ZPs) quickly lost their antioxidant activity (*P* < 0.05). In addition, free CEO did not contribute any antioxidant activity to the water matrix. This release‐related antioxidant activity indicated that CEO may be only able to participate in the proposed DPPH scavenging when encapsulated. In skimmed and whole milk, as shown in Fig. 3(B),(C), the addition of free CEO did not improve the overall DPPH radical scavenging activity, although the antioxidant activity of free CEO has been widely reported^27^ and a significant amount of free CEO was able to dissolve into milk matrices (Fig. 2(B),(C)). The dissolved CEO was most likely sequestered from the DPPH radicals by the hydrophobic milk components, preventing its participation in the radical quenching reaction.^28^ Additionally, the abundant proteins and fats in milk compete for DPPH radical scavenging due to their inherent antioxidant properties (Supporting Information, Fig. S1), which may mask the potential antioxidant contribution of CEO that is accessible to radicals. Food matrices with complex compositions, such as milk containing proteins and fats, impose greater demands on antioxidants requiring high accessibility and reactivity. Nevertheless, even amid this interference from antioxidative milk components, encapsulated CEO, particularly the slow‐release formulations, demonstrated superior apparent antioxidant performance compared to free CEO. As shown in Fig. 3(B),(C), the antioxidant ability of CaP‐AlgGel‐CEO/ZPs was evident throughout the storage period (*P* < 0.05), while CEO/ZPs and Alg‐CEO/ZPs only demonstrated considerable antioxidant activity at the first two timepoints (*P* < 0.05). Encapsulated CEO was more accessible to DPPH radicals than the milk‐dissolved CEO, probably attributed not only to the isolation of CEO from the milk components but also to the ability of zein‐based encapsulates to dissolve in DPPH ethanolic solution,^29^ which facilitated CEO release for scavenging DPPH radicals. Other common antioxidant assays, such as ABTS, FRAP, and ORAC, may yield different results due to the absence of such a release mechanism and their different underlying principles.^30^ Thus, a comprehensive evaluation of the antioxidant capacity of CEO encapsulates should incorporate multiple assessment assays in future studies. Despite this, the current DPPH results underscored one of those potentials of encapsulation strategies to enhance the accessibility of bioactive compounds like CEO to some oxidative factors within complex food matrices.

### Antibacterial effect of CEO encapsulates in matrices

**Figure 4:**
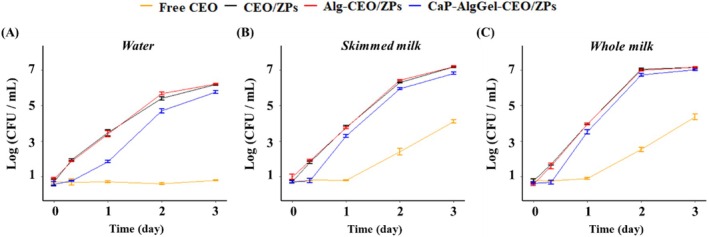
Changes in bacterial counts in (A) water, (B) skimmed milk, and (C) whole milk during storage at 25 °C for 3 days after mixing with CEO or CEO encapsulates. CEO/ZPs: citrus essential oil‐encapsulated zein particles; Alg‐CEO/ZPs: alginate‐supplemented CEO/ZPs; CaP‐AlgGel‐CEO/ZPs: calcium phosphate‐coated and alginate gel‐networked CEO/ZPs.

In this study, total aerobic mesophilic bacteria were enumerated over time in the matrices. The initial bacterial counts in pasteurized skimmed and whole milk were very low, measured as single‐digit colony‐forming units (CFU) per milliliter. Only a small number of thermoduric bacteria typically survive pasteurization, and any post‐pasteurization contamination is often attributable to psychrotrophic bacteria.^31^ Following the supplementation of free CEO or CEO encapsulates, the growth of bacteria in food matrices was influenced by both the antibacterial properties of the CEO additives and the availability of nutrients. As shown in Fig. 4(A), bacterial growth was absent in water when supplemented with free CEO, while zein‐based encapsulates (i.e., CEO/ZPs, Alg‐CEO/ZPs, and CaP‐AlgGel‐CEO/ZPs) provided sufficient nutrients to support bacterial proliferation. This difference in nutrient availability also resulted in significant variations in bacterial growth across different milk matrices, with and without the addition of zein‐based encapsulates (Fig. 4(B),(C)). However, under the same nutrient levels, bacterial growth rates of CEO capsules with different release profiles within a specific food matrix were comparable. The growth data were well‐fitted to a modified Gompertz model through non‐linear regression (Table 2). Across all three matrices, the viable bacterial count was significantly lower in the presence of CaP‐AlgGel‐CEO/ZPs before reaching the asymptotic population compared to the other two CEO encapsulates (*P* < 0.05) (Fig. 4). This observation was closely associated with the significantly prolonged lag phases (*λ*) (*P* < 0.05) observed with CaP‐AlgGel‐CEO/ZPs supplementation, which hindered the bacterial population growth, outweighing the positive effect of their relatively higher *μ*_max_. In addition, the *A*_s_ with CaP‐AlgGel‐CEO/ZPs supplementation was slightly lower than that with CEO/ZPs and Alg‐CEO/ZPs, further indicating the greater bacterial inhibition with CaP‐AlgGel‐CEO/ZPs supplementation. The antibacterial ability of CaP‐AlgGel‐CEO/ZPs could be attributed primarily to the slower CEO‐releasing rate, because the insignificant bacterial growth between CEO/ZPs and alginate supplemented ZPs (namely Alg‐CEO/ZPs) suggested that alginate does not significantly affect bacterial growth. Also, studies have further shown that calcium alginate, when not incorporating antibacterial agents, exhibited no meaningful antibacterial properties.^32^, ^33^, ^34^ It has been reported that encapsulation prevents the interaction of essential oils with milk components such as carbohydrates, proteins, and lipids, thereby increasing the bioavailability of essential oils for pathogens.^13^ Within the same matrices, slow CEO‐releasing encapsulates were superior to others, indicating that the temporal profile of CEO availability is more critical than the total dissolved amount. Given that CEO exhibits a broad‐spectrum antibacterial activity encompassing common food spoilage bacteria,^35^ the involved experiment at 25 °C had the instructive meaning as an accelerated experiment for low‐temperature storage conditions, even though the microbial community in pasteurized milk may differ between these two storage temperatures.^31^

**Table 2:** The natural logarithm of the asymptotic value of relative population size (*A*_s_), the maximum specific growth rate (*μ*_max_), the duration of the lag phase (*λ*), and adjusted *R* ^2^ as calculated from a modified Gompertz model fitted to the plate counts in citrus essential oil (CEO) encapsulate‐supplemented matrices

		Water			Skimmed milk			Whole milk	
	CEO/ZPs	Alg‐CEO/ZPs	CaP‐AlgGel‐CEO/ZPs	CEO/ZPs	Alg‐CEO/ZPs	CaP‐AlgGel‐CEO/ZPs	CEO/ZPs	Alg‐CEO/ZPs	CaP‐AlgGel‐CEO/ZPs
*A*_s_ (ln(CFU mL^−1^))	12.96 ± 0.60^e^	12.95 ± 0.46^de^	12.61 ± 0.40^de^	15.51 ± 0.42^b^	14.92 ± 0.40^ab^	14.15 ± 0.31^cd^	15.47 ± 0.39^ab^	15.9 ± 0.42^a^	14.77 ± 0.23^bc^
*μ*_*max*_ (d^−1^)	6.69 ± 0.68^d^	6.93 ± 0.56^cd^	7.52 ± 0.52b^cd^	8.17 ± 0.48b^cd^	8.09 ± 0.49^bcd^	10.16 ± 0.81^bc^	10.14 ± 0.77^b^	10.34 ± 0.8^b^	15.11 ± 1.99^a^
*λ* (day)	0.05 ± 0.08^c^	0.12 ± 0.06^c^	0.62 ± 0.05^a^	0.13 ± 0.05^c^	0.21 ± 0.05^c^	0.43 ± 0.05^b^	0.23 ± 0.05^c^	0.2 ± 0.05^c^	0.56 ± 0.06^ab^
Adjusted *R* ^2^	97.53%	98.54%	99.42%	99.24%	99.26%	99.42%	99.04%	98.96%	99.67%

*Note*: The superscript lowercase letters in a row indicate the significant differences (*P* < 0.05). CEO/ZPs: CEO‐encapsulated zein particles (ZPs); Alg‐CEO/ZPs: alginate‐supplemented CEO/ZPs; CaP‐AlgGel‐CEO/ZPs: calcium phosphate‐coated and alginate gel‐networked CEO/ZPs.

### Relation between CEO distribution and matrix quality

**Figure 5:**
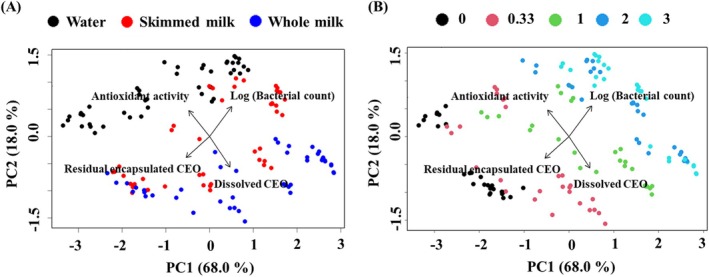
Principal component analysis (PCA) loading plot for residual encapsulated CEO in CEO encapsulates, dissolved CEO in food matrices, antioxidant activity of CEO encapsulates, and logarithm of bacterial count in matrices, in combination with PCA score plots that were labeled by information of (A) matrices and (B) time points (day), respectively.

A PCA was performed on the four attributes measured in CEO encapsulate‐supplemented matrices. These attributes included residual encapsulated CEO in CEO encapsulates, dissolved CEO in food matrices, antioxidant activity of CEO encapsulates, and logarithm of bacterial count in matrices. The loading plot in Fig. 5 displays the correlations between the variables in an unsupervised manner. It was revealed that the antioxidant activity of encapsulated CEO and logarithm of the bacterial count was negatively correlated with the CEO solubilization and CEO that remain encapsulated, respectively. These findings were aligned with the conclusions drawn from the previous sections. Specifically, dissolved CEO was almost ineffective in scavenging DPPH radicals, whereas residual encapsulated CEO was effective in inhibiting bacterial growth, resulting in a lower bacterial count.

In Fig. 5(A),(B), the matrices and time points are labeled, respectively. A clear group clustering was observed, indicating the significant influence of both matrix type and storage time on the properties of the ZP–matrix mixtures. The projections of sample points onto the loading vectors revealed that the matrix types primarily influenced the antioxidant activity of CEO encapsulates and CEO solubilization, while storage time had a greater influence on bacterial growth in matrices and residual encapsulated CEO.

## CONCLUSIONS

Water‐insoluble CEO was able to dissolve in milk matrices due to strong interactions with hydrophobic milk components. However, these interactions impair the CEO's ability to scavenge DPPH radicals, as the hydrophobic bindings sequestered the CEO and diminished its antioxidant capacity. Encapsulating CEO in ZPs with a slower release rate, such as CaP‐AlgGel‐CEO/ZPs, improved these functions by prolonging the encapsulation period of CEO, thereby reducing undesirable interactions with fatty milk components. In comparison with water, milk enhances the release of CEO from ZPs through increased diffusion forces. The released CEO gradually dissolves in the milk matrices, aided by the solubilization facilitated by the high fat content. Moreover, milk components could serve as colloidal stabilizers for CEO, enhancing its solubility, particularly when the encapsulates were at the nanoscale. PCA identified matrix type and storage time as two significant factors influencing the quality of CEO encapsulate‐supplemented matrices. The type of matrix affects the antioxidant activity of the CEO encapsulates, while storage time impacts their antibacterial effectiveness. This study highlights the benefits of incorporating encapsulated bioactive substances in food matrices to enhance their functional properties. Encapsulation techniques present a viable solution for mitigating the interactions between bioactive substances and food components by providing a protective separation. The effectiveness of these techniques will depend on the careful design of the specific encapsulated ingredient tailored to specific food matrices.

## CONFLICT OF INTEREST

The authors declare no competing financial interest.

## AUTHOR CONTRIBUTIONS

Feilong Yang: conceptualization; methodology; investigation; data analysis; visualization; draft writing. Vincenzo Fogliano: conceptualization; funding acquisition; supervision; review and editing. Ashkan Madadlou: conceptualization; methodology; validation; supervision; review and editing.

## Supporting information


**Figure S1.** DPPH radical scavenging activity (%) of water, skimmed milk, and whole milk during 3‐day storage.

## Data Availability

The data that support the findings of this study are available on request from the corresponding author. The data are not publicly available due to privacy or ethical restrictions.
