# Supplementary material for: The effect of citrus essential oil encapsulation on antioxidant capacity and bacterial growth in a milk matrix during storage
Source: J Sci Food Agric. 2025 Nov 20;106(4):2352–60. doi: 10.1002/jsfa.70344 (PMC12872250; doi:10.1002/jsfa.70344)
Supplement: Supplementary file 1 — Figure S1. DPPH radical scavenging activity (%) of water, skimmed milk, and whole milk during 3‐day storage. [file JSFA-106-2352-s001.docx]

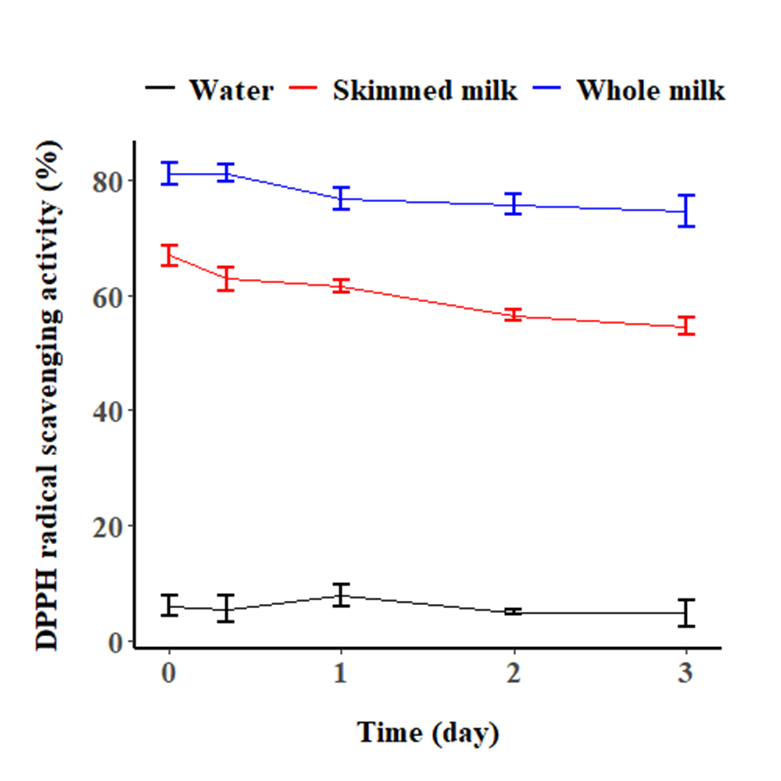


Supplementary Fig. 1. DPPH radical scavenging activity (%) of water, skimmed milk, and whole milk during 3-day storage.
